# Low temperature and cost-effective growth of vertically aligned carbon nanofibers using spin-coated polymer-stabilized palladium nanocatalysts

**DOI:** 10.1088/1468-6996/16/1/015007

**Published:** 2015-02-25

**Authors:** Amin M Saleem, Sareh Shafiee, Theodora Krasia-Christoforou, Ioanna Savva, Gert Göransson, Vincent Desmaris, Peter Enoksson

**Affiliations:** 1 Smoltek AB, Regnbågsgatan 3, Gothenburg, SE-41755, Sweden; 2 Micro and Nanosystems group, BNSL, Department of Microtechnology and Nanoscience, Chalmers University of Technology, Gothenburg, SE-41296, Sweden; 3 Department of Mechanical and Manufacturing Engineering, University of Cyprus, Nicosia, Cyprus; 4 Nanotechnology Research Center (NRC), University of Cyprus, Nicosia, Cyprus; 5 Department of Chemistry and Molecular Biology, University of Gothenburg, Gothenburg, SE-41296, Sweden

**Keywords:** polymer-stabilized nanoparticles, carbon nanofibers, low temperature growth, cost effective, carbon, 81.05.U-, carbon nanotubes, 88.30.rh, chemical vapor deposition, 81.15.Gh, polymers, 81.05.Qk, micro- and nanofabrication, 81.16.-c, Nanoscale materials and structures, 81.07.-b

## Abstract

We describe a fast and cost-effective process for the growth of carbon nanofibers (CNFs) at a temperature compatible with complementary metal oxide semiconductor technology, using highly stable polymer–Pd nanohybrid colloidal solutions of palladium catalyst nanoparticles (NPs). Two polymer–Pd nanohybrids, namely poly(lauryl methacrylate)-block-poly((2-acetoacetoxy)ethyl methacrylate)/Pd (LauMA_*x*_-b-AEMA_*y*_/Pd) and polyvinylpyrrolidone/Pd were prepared in organic solvents and spin-coated onto silicon substrates. Subsequently, vertically aligned CNFs were grown on these NPs by plasma enhanced chemical vapor deposition at different temperatures. The electrical properties of the grown CNFs were evaluated using an electrochemical method, commonly used for the characterization of supercapacitors. The results show that the polymer–Pd nanohybrid solutions offer the optimum size range of palladium catalyst NPs enabling the growth of CNFs at temperatures as low as 350 °C. Furthermore, the CNFs grown at such a low temperature are vertically aligned similar to the CNFs grown at 550 °C. Finally the capacitive behavior of these CNFs was similar to that of the CNFs grown at high temperature assuring the same electrical properties thus enabling their usage in different applications such as on-chip capacitors, interconnects, thermal heat sink and energy storage solutions.

## 1. Introduction

Carbon nanotubes (CNTs) were discovered by Sumio Iijima in 1991 [1] and since then, they have received high attention due to their extraordinary mechanical, electrical and thermal properties. Besides that, CNTs have very low density [2] and very high aspect ratio. CNTs consist of graphene sheets coaxially rolled into hollow cylinders, and can be single-walled or multi-walled depending on the number of graphene sheets. Moreover, CNTs can be metallic or semiconducting depending on the twist of the tubes.

In contrast to CNTs, carbon nanofibers (CNFs) comprise cone-shaped graphene layers stacked on top of each other creating completely filled cylinders, which also have excellent mechanical, thermal and electrical properties. At 400 and 7 GPa, the Young modulus and tensile strength of CNFs are higher than those of steel [3]. The thermal conductivity of CNFs (about 1900 W mK^−1^) is five times higher than copper [4] and can carry current density of about 12 MA cm^−2^ which is three times higher than copper [5, 6]. CNFs exhibit many significant advantages over CNTs, since they can be grown at lower temperatures and they are 100% metallic, whereas 2/3 of CNTs are semiconducting when grown in bulk [7]. Because of these excellent properties CNFs have potential applications in electrical interconnects [8], as thermal interface materials, as reinforcement additives in polymer composites and as electrodes for supercapacitors.

The carbon nanostructures, including CNFs, can be synthesized in several ways including electrospinning, chemical vapor deposition (CVD), laser ablation and arc discharge. There are different types of CVD and these are classified on the basis of energy type used during deposition: microwave plasma enhanced CVD, thermal CVD and direct current plasma enhanced CVD (DC-PECVD) [9–11]. Depending on the catalyst and the CVD growth method used, different types of carbon nanostructures are obtained. The DC-PECVD method has an advantage over the other growth methods in that it provides high purity and vertical alignment of the produced CNFs [12].

To grow CNFs using CVD, the deposition of catalytic particles on a substrate is required. Transition metals such as iron, cobalt, palladium and nickel are often used as catalysts [11, 13–15]. The catalysts can be deposited in different ways onto the substrate such as deposition of a pure metal film using sputtering or e-beam evaporation techniques and deposition of a nanoparticle (NP)-containing solution via spin coating, dip-coating or spraying methods [16–18]. The physical vapor deposition (PVD) deposition methods, such as sputtering or evaporation, require expensive equipment, a metal target, clean room laboratory facilities and costly equipment maintenance.

In the present study, catalytic palladium NPs are deposited on silicon substrate via spin-coating using highly stable and inexpensive polymer–Pd colloidal solutions. CNFs are then grown on these catalyst particles at complementary metal oxide semiconductor (CMOS) process-compatible temperature thus allowing CNFs to be used in on-chip thermal, electrical and energy storage solutions.

Two types of polymer–Pd NPs solutions were used to deposit the palladium catalyst particles: (1) poly(vinylpyrro1idone)-Pd (PVP–Pd) NPs stabilized in methanol solution and (2) poly(lauryl methacrylate)-block-poly(2-(acetoacetoxy)ethyl methacrylate) (LauMA_*x*_-b-AEMA_*y*_/Pd) micellar nanohybrids stabilized in *n*-hexane. Different types of polymers are used due to their different thermal stabilization temperatures. The polymers are stable up to certain temperature and burn off when heated to higher temperature thus leaving Pd NPs on the surface of the substrate. The stabilization temperature of LauMA_*x*_-b-AEMA_*y*_ and PVP polymers is around 200 °C and 400 °C respectively [19]. The synthesis and characterization of the aforementioned polymer-stabilized Pd NPs has been reported in other studies [20–23]; however, in this work these polymer-metal nanohybrids have been employed for the growth of CNFs. The use of such catalyst-containing solutions is advantageous since those are nontoxic, cheap and can be easily prepared using low-cost wet-chemistry procedures. In addition, their deposition onto selected substrates does not require the use of expensive equipment. Moreover, the polymers can be easily washed away and the substrate can be reused in case of process failure during NPs deposition. This is a rapid and industry compatible deposition method that can be used to grow a film of CNFs at the backside of a chip for heat sink applications.

Three different PVP–Pd nanohybrid solutions were prepared in methanol, in which the polymer content was kept constant and only the palladium content varied. In the case of the LauMA_*x*_-b-AEMA_*y*_/Pd micellar systems stabilized in *n*-hexane, two different block copolymers (polymer A: *x* = 120, *y* = 67; polymer B: *x* = 50, *y* = 9) are employed as the palladium NPs stabilizing agents. The CNFs were grown using the DC-PECVD technique at different temperatures i.e. at a high temperature (550 °C), at a CMOS compatible temperature (390 °C) and even at 40 °C lower (350 °C). The specific capacitance and cyclability of the CNFs were measured using cyclic voltammetry to demonstrate the electrical properties and applicability of these nanostructures as supercapacitor electrodes directly built on CMOS chips.

## 2. Fabrication, characterization and measurements

The synthetic methodologies followed for the preparation of the PVP–Pd and the LauMA_*x*_-b-AEMA_*y*_-Pd hybrid solutions are described below.

(1) A typical synthetic methodology followed for the synthesis of the PVP–Pd nanohybrid colloidal systems (moles vinyl pyridine units/moles palladium salt = 9:1) is described as follows [19, 23]: in a round bottom flask equipped with a magnetic stirrer, PVP (1 g, 9 × 10^−3^ moles of vinylpyrridine units) (Sigma-Aldrich, MW = 1300 000) was dissolved in methanol (10 mL). Subsequently, palladium acetate (0.22 g, 1 × 10^−3^ moles) (Pd(OAc)_2_, Sigma-Aldrich, 98%) was added to the polymer solution and the reaction flask was placed under reflux for 2 h. During this period, the color of the solution changed from yellow to dark brown indicating the formation of palladium NPs. After the completion of the reaction, the brown-colored solution was left to cool down to room temperature and it was then stored in sealed glass vials. The solutions were highly stable and no precipitation was observed even after several months.
  The above-mentioned methodology was employed for synthesizing two more PVP–Pd nanohybrid colloidal systems stabilized in methanol, in which the polymer content was kept the same and only the metallic (Pd) content varied i.e. the molar ratio of the vinyl pyridine units to the palladium salt was 18:1 and 38:1.
(2) The Pd-containing LauMA_*x*_-b-AEMA_*y*_ micellar hybrids have been prepared in *n*-hexane by following a previously reported methodology [20, 22]. Exemplarily, the procedure followed for the synthesis of the LauMA_120_-b-AEMA_67_/Pd micellar nanohybrids is described as follows: LauMA_120_-b-AEMA_67_ (20 mg, 0.03 mmoles of AEMA units) was dissolved in *n*-hexane (5 mL). After complete dissolution of the polymer, triethylamine (0.021 mL, 0.15 mmoles ) was added. Subsequently, the polymer solution was transferred into a glass vial containing Pd(OAc)_2_ (3.40 mg, 0.015 mmoles) and the mixture was left to stir at room temperature until complete solubilization of the salt. Upon complexation and solubilization, the color of the solution changed from white to yellow transparent. Finally, the reducing agent namely hydrazine monohydrate (1.46 *μ*L, 0.03 mmoles) was added to the solution upon stirring. This was accompanied by a color change of the solution from yellow to dark brown indicating the reduction of palladium (II) ions into metallic palladium (0) NPs. The resulting solution was left to stir for 24 h at room temperature. The size of the palladium NPs generated within the micellar cores has been already reported [22]. The average diameter of the palladium NPs is 2.3 ± 0.3 nm for the LauMA_50_-b-AEMA_9_/Pd(solution A) and 8.8 ± 1.7 nm for LauMA_120_-b-AEMA_67_/Pd(solution B).

A 2 inch n-type silicon wafer was used as a substrate for the uniform deposition of the polymer-palladium solutions. Titanium/titanium nitride (Ti/TiN) of thicknesses 50 and 100 nm were sputtered on each side of the chip using FHR MS 150 sputter machine in order to have better electrical contact between back and front side of the sample, because of backside probing of the chip for measurements. The polymer-palladium NPs solutions were later spun using standard resist spinners. Vertically aligned CNFs were grown at different temperatures by the DC-PECVD method as described in [8, 24]. A mixture of acetylene and ammonia gases was used where the acetylene is the source gas and the ammonia is a carrier gas. The growth was executed for 2 h on all samples. The temperature of the heater is measured and regulated during the whole growth process to an accuracy of ±2° using a built-in thermocouple inside the grounded heating plate.

The chips were weighed before the deposition of the palladium NPs and after the growth of CNFs for determining the weight of the CNFs including catalyst, a parameter used in evaluating the capacitance per gram of such electrodes. The weight measurements were performed using a high precision balance ‘Sartorius Analytical Balance BP211D’ with a resolution of 10 *μ*g. Transmission electron microscopy (TEM) analysis performed on the LauMA_*x*_-b-AEMA_*y*_/Pd (solution A) system was carried out on a 1010 JEOL microscope (200 kV). The suspension of MNPs was dried on a carbon coated copper grid to allow the TEM investigation. The scanning electron microscopy (SEM) analysis of the CNFs was conducted using JEOL JSM-6301F. In order to see the morphology of the CNFs, images were taken at 40° tilt angle as well as top view images at zero tilt.

Cyclic voltammetry (CV) was performed to evaluate the capacitance. A three electrode setup was used with a silver/silver chloride (Ag/AgCl) electrode as the reference electrode and platinum mesh as the counter electrode. The electrolyte used was 1M KOH (99.99%, Aldrich). The limit of measuring the electrode cell was one centimeter diameter disk. The samples were mounted and dipped in the electrolyte solution for a few minutes while nitrogen gas was blowing to wet the CNFs. The CV curve was obtained using Gamry Instruments Reference 600 (Framework 6.1). The voltage sweep ranging from −0.2 to 0.3 V was applied and different voltage scan rates were used. The voltammetry using 10 mV s^−1^ scan rate was carried out for 18 cycles to exclude the doubt of initial surface reactions whereas 5 cycles were run for other scan rates. The same measurement was also performed for 1000 cycles to measure the cyclability at 20 mV s^−1^ scan rate while keeping the other parameters the same. The ideal double layer capacitance behavior is the rectangular shape of the voltammetry characteristics drawn between current and voltage [25].

## 3. Results and discussion

Information on the palladium NPs content (provided as the molar ratio of the metal-binding units of the polymer to the palladium salt precursor), growth temperature, weight of CNFs and capacitance is provided in table 1, whereas the specific capacitance measured at different voltage scan rates is given in table 2.

**Table 1.** Properties of the CNFs grown using different polymer-palladium NPs solutions at different temperatures, their capacitance and specific capacitance mF cm^−2^ (per footprint area).

[vinyl pyridine]/[Pd(OAc)_2_]	Growth temperature (°C)	Weight (*μ*g)	Length (*μ*m)	Capacitance (mF)	Specific capacitance (mF cm^−2^)
Bare chip				0.1	0.12
38:1	550	53	14	0.8	1
18:1	550	245	9	3.1	4
9:1	550	916	5	7.1	9
9:1	390	612	1	2.6	3.3
[AEMA]/[Pd(OAc)_2_]					
[LauMA_120_-b-AEMA_67_] 2:1	390	654	1	2.1	2.7
[LauMA_50_-b-AEMA_9_] 2:1	390	115	2	1.4	1.8
[LauMA_120_-b-AEMA_67_] 2:1	350	250	1.4	3.1	4
[LauMA_50_-b-AEMA_9_] 2:1	350	115	1.6	0.2	0.2

**Table 2.** Specific capacitance mF cm^−2^ (per footprint area) measured at different voltage scan rates.

[vinyl pyridine]/[Pd(OAc)_2_]	*T* (°C)	10 mV s^−1^	20 mV s^−1^	50 mV s^−1^	100 mV s^−1^
PVP/Pd 38:1	550	1	0.8	0.7	0.6
PVP:Pd 18:1	550	4	2.4	1.6	1.2
PVP/Pd 9:1	550	9.	6	4.6	4
PVP/Pd 9:1	390	3.3	2.6	2	2
[AEMA]/[Pd(OAc)_2_]					
Solution A	350	0.2	0.2	0.15	0.14
Solution B	390	2.7	2	1.5	1.3
Solution B	350	4	3	2.3	2
Solution A	390	1.8	1.41	1.1	1

**Figure 1.**
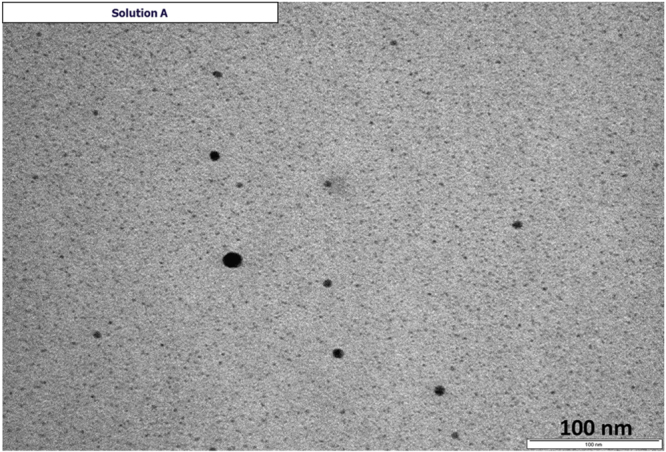
TEM image of the LauMA_50_-b-AEMA_9_/Pd system (solution A).

**Figure 2.**
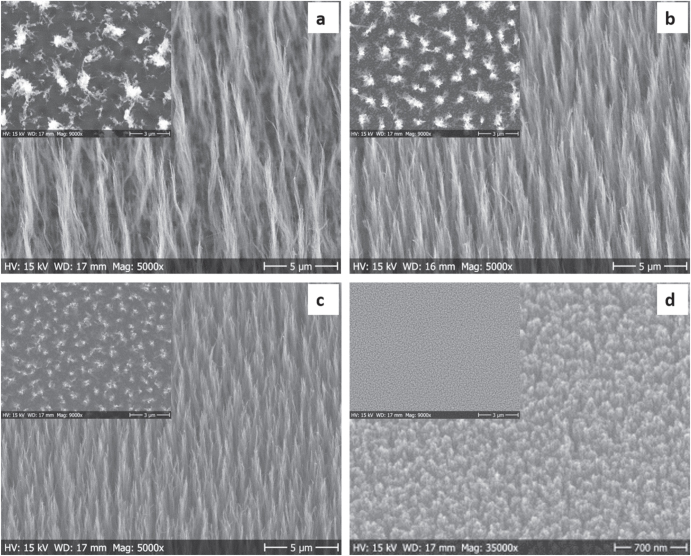
SEM images of CNFs grown by DC-PECVD for 2 h at different temperatures on palladium NPs using various PVP–Pd colloidal solutions given as (a) PVP:Pd 38:1, growth at 550 °C. (b) PVP:Pd 18:1, growth at 550 °C. (c) PVP:Pd 9:1, growth at 550 °C. (d) PVP:Pd 9:1, growth at 390 °C.

A representative TEM image of the solution A system is provided in figure 1. As seen in the image, tiny, spherical palladium NPs can be visualized with average diameters in the range 2.3 ± 0.3 nm. The presence of a few larger aggregates may be due to drying-induced aggregation during TEM sample preparation. In the case of the PVP/Pd systems, HRTEM analysis revealed that the palladium NPs are nanocrystals with average diameters below 10 nm. The crystalline planes (111) and (200) of palladium NP could be visualized with characteristic interplanar distances of 2.27 and 1.97 Å, respectively [26]. Tilted view and top view (inset on each picture) SEM images of CNFs grown at 550 °C using three PVP/Pd solutions having different metallic (palladium) content are shown in figures 2(a)–(c) whereas SEM images of the CNFs grown at 390 °C using the PVP/Pd (1:9) solution is provided in figure 2(d).

**Figure 3.**
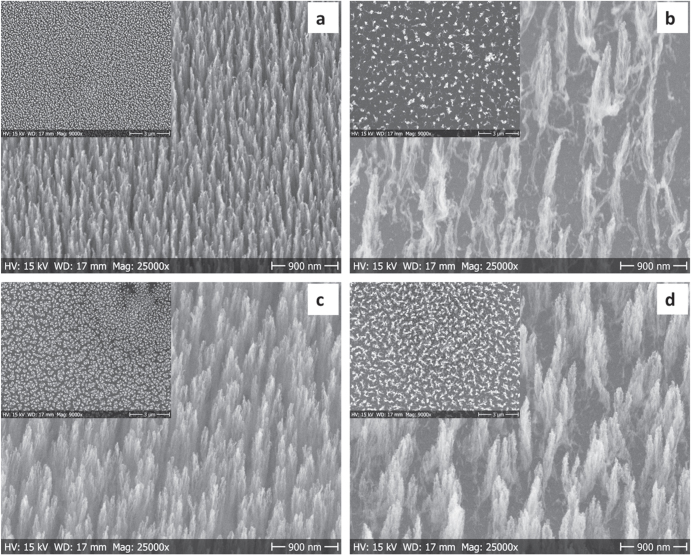
SEM images of CNFs grown by DC-PECVD for 2 h on two different LauMA_*x*_-b-AEMA_*y*_/Pd micellar solutions at different temperatures given as (a) LauMA_120_-b-AEMA_67_/Pd (solution B) growth at 390 °C. (b) LauMA_50_-b-AEMA_9_/Pd (solution A) growth at 390 °C. (c) LauMA_120_-b-AEMA_67_/Pd (solution B) growth at 350 °C. (d) LauMA_50_-b-AEMA_9_/Pd (solution A) growth at 350 °C.

Similarly, the tilted view and top view (inset on each picture) SEM images of the CNFs grown at 390 and 350 °C using solution A and solution B are shown in figures 3(b), (d) and (a), (c).

It is clear from the SEM images that the CNFs grown at lower temperature 350 °C are vertically aligned similar to those grown at the highest temperature (550 °C) as shown in figures 2 and 3. In addition, inset of figures 2 and 3 also show the uniform growth of CNFs confirming the uniform deposition of palladium catalyst NPs using spin-coating, which is in line with [26].

Moreover, in the case of the PVP–Pd systems, the CNF density increases significantly by increasing the palladium content within the PVP–Pd colloidal hybrid solutions i.e. from 38:1 to 18:1 and to 9:1 (figures 2(a)–(c) respectively) thus resulting in an increase in CNF weight, as shown in table 1. In addition, SEM analysis also reveals that under identical growth conditions, the CNFs grown using the highest palladium-containing system (9:1) are shorter in length compared to those grown using the lowest palladium-containing system (38:1). By using the same palladium-containing system (9:1) the CNFs grown at higher (550 °C) temperatures are much longer than those grown at 390 °C, in line with the weight measurements, although less dense than the CNFs grown at 390 °C.

In the case of the LauMA_*x*_-b-AEMA_*y*_/Pd solutions, the average diameter of the palladium nanocatalyst is 2.3 ± 0.3 nm for solution A and 8.8 ± 1.7 nm for solution B. Despite their size differences both systems are found within the optimum size range enabling CNF growth at a low temperature (350 °C) as seen in the SEM images provided in figures 3(c) and (d). The top view SEM images (inset in figure 3) clearly show that under identical growth temperature, the CNFs grown using solution B are denser and heavier than those grown using solution A, but shorter in length compared to those obtained from solution A, as shown in table 1. Furthermore, when the growth is performed at different temperatures, solution B gives longer but less dense CNFs at low temperature 350 °C. On the other hand, solution A gives denser and shorter CNFs at the same growth temperature.

**Figure 4.**
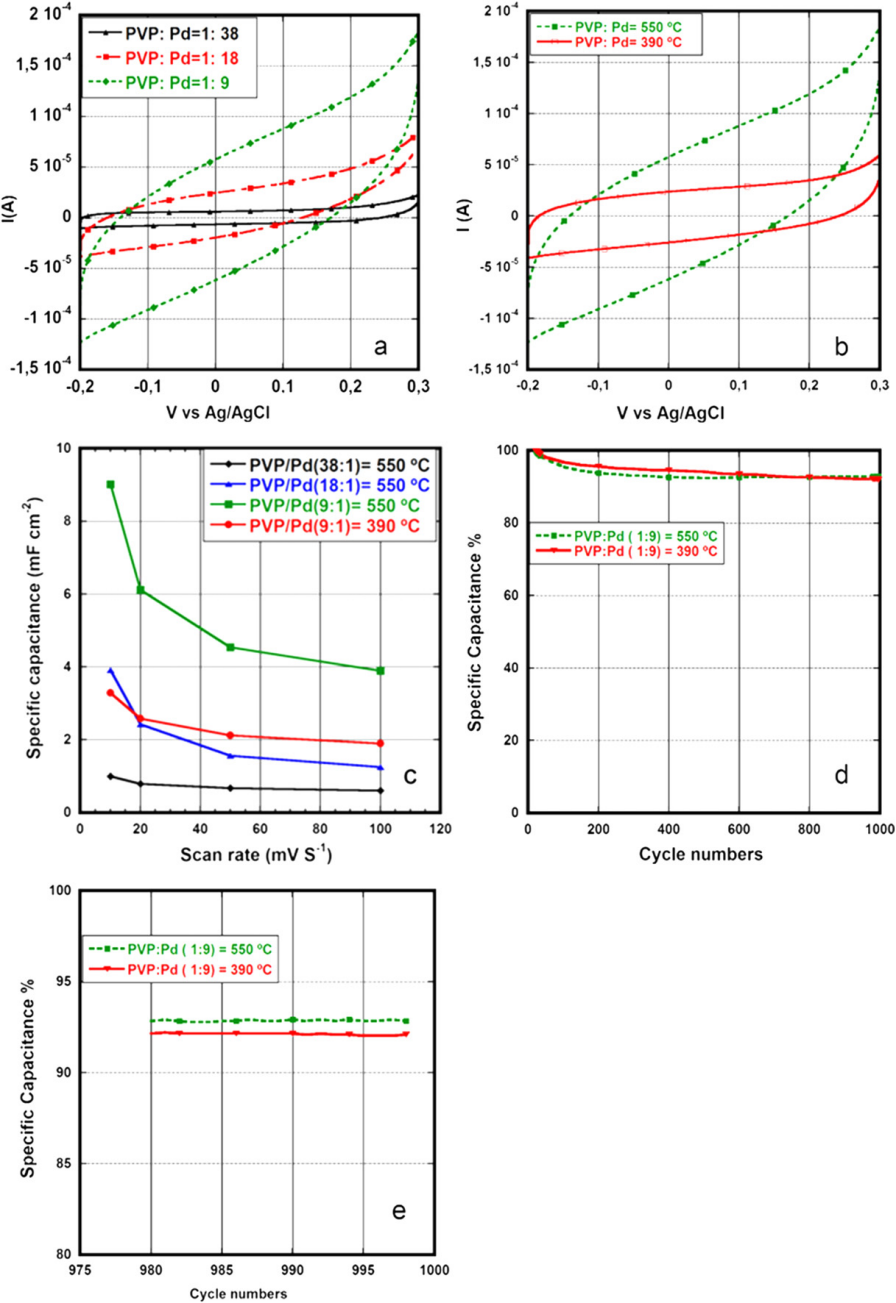
Cyclic voltammetry curves of CNFs. (a) At 550 °C using different PVP–Pd solutions with different palladium content. (b) At 550 °C and 390 °C using PVP:Pd 9:1solution. (c) Specific capacitance (mF cm^−2^ foot print area) versus voltage scan rate. (d) Cyclability of CNFs grown at 550 °C and 390 °C using PVP:Pd 9:1 solution (normalized by specific capacitance of first cycle). (e) Cycles from 975 to 999.

**Figure 5.**
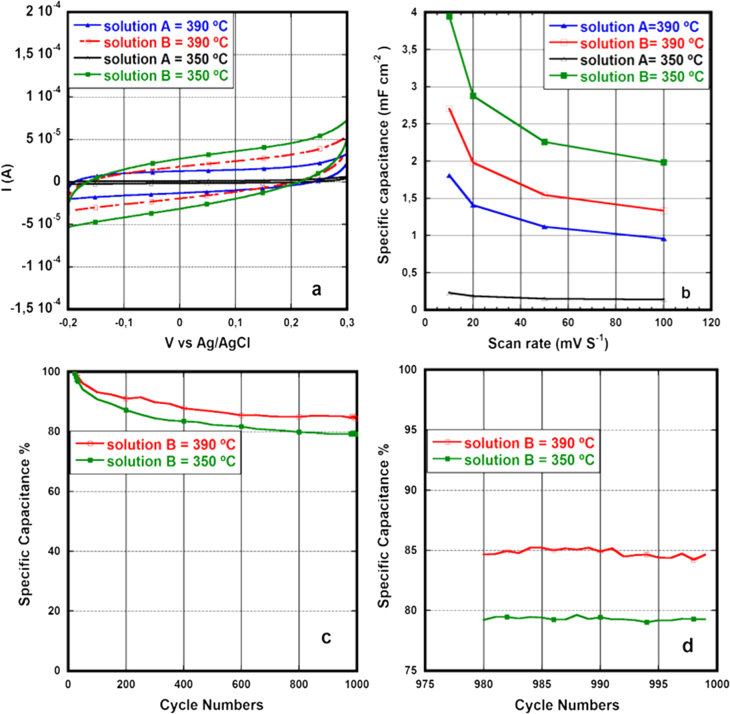
(a) Cyclic voltammetry curves of CNFs grown using solution A and solution B at 350 °C and 390 °C. (b) Specific capacitance (mF cm^−2^ foot print area) versus voltage scan rate. (c) Cycle life of solution B (normalized by specific capacitance of first cycle). (d) Cycles from 980 to 999.

The CV curves for the electrical characterization of the CNFs grown using the PVP–Pd and the LauMA_*x*_-b-AEMA_*y*_-Pd solutions are provided in figures 4(a), (b) and 5(a). The specific capacitance (mF cm^−2^ footprint area) curves measured at difference voltage scan rate are shown in figures 4(c) and 5(b). The cyclability curves for 1000 cycles are shown in figures 4(d) and 5(c) whereas magnified images of the last 20 cycles from 980 to 999 are shown in figures 4(e) and 5(d).

The voltammograms given in figures 4(a), (b) and 5(a) are fairly rectangular showing the double layer capacitive behavior of the CNFs obtained using both types of polymer–Pd catalyst solutions, however, the peaks at extreme sweep voltages show some pseudocapacitive charge storage which may arise from the reaction of underlayer with aqueous electrolyte.

At 550 °C growth temperature and 10 mV s^−1^ scan rate, the specific capacitance (per cm^−2^ foot print area) is larger (9 mF cm^−2^) for higher concentration (9:1) of palladium NPs in the PVP–Pd solution and lower (1 mF cm^−2^) for lower concentration (38:1) confirming the active capacitive role of CNFs. In fact, shorter but denser CNFs for the (9:1) solution give a higher surface area accessible to electrolyte than longer but less dense CNFs for the (38:1) solution, and the higher surface area results in higher current on voltammogram and hence higher specific capacitance as shown in figure 4(a) and table 1. In addition the specific capacitance is 3.3 mF cm^−2^ for the CNFs grown at 390 °C using the PVP–Pd (9:1) solution.

In the case of the LauMA_*x*_-b-AEMA_*y*_/Pd solutions the specific capacitance is higher (2.7 and 4 mF cm^−2^) for CNFs synthesized using solution B than solution A when grown at corresponding temperatures 350 °C and 390 °C which is in line with the weight of the CNFs. Moreover, for the same solution, the longer CNFs give a higher surface area which results in higher current on the voltammogram and hence higher specific capacitance, as shown in figure 5(a) and table 1. The capacitive behavior indicates the similar electrical behavior of CNFs grown at both low and high temperatures.

The specific capacitance at 100 mV s^−1^ ranges from 3.9 to 0.140 mF cm^−2^. This specific capacitance is many folds higher than the recently reported capacitance obtained from the CVD grown vertically aligned CNFs [27]. Furthermore, the maximum specific capacitance at 100 mV s^−1^ scan rate is 3.9 mF cm^−2^ obtained from the CNFs grown at 550 °C, table 2, which is almost equal to the maximum reported capacitance obtained from graphene-CNT based microcapacitors (3.93 mF cm^−2^) synthesized on a similar silicon substrate but using a longer voltage scan range [28]. Moreover, the specific capacitance obtained from CNFs grown at low temperature 350 °C and 390 °C, table 2, is 1.9–2 mF cm^−2^, half of the reported specific capacitance mentioned above, nevertheless the growth temperature of CNFs here is below the CMOS process-compatible temperature. In addition, the cyclic voltammetry at different voltage scan rates shows that specific capacitance decreases by increasing the scan rate from 10 to 100 mV s^−1^ for both polymer–Pd types used, figures 4(c) and 5(b), due to a kinetically slow faradaic reaction on the electrode surface which does not take place at high scan rate^5^ 5www.gamry.com/application-notes/testing-electrochemical-capacitors-part-1-cyclic-voltammetry-and-leakage-current/, accessed successfully on 06-10-2014..

Moreover, the cycle life curves of both polymer–Pd solution types show the decrease in specific capacitance with increase in cycles, figures 4(d) and 5(c), which eventually becomes uniform and remains unchanged as shown in the plots of the last 20 cycles, given in figures 4(e) and 5(d). The more than 90% capacitance retention for CNFs grown from the PVP–Pd solution and 80% for the CNFs grown from solution B after 1000 cycles of cyclic voltammetry proves the long cycle life of these materials as supercapacitor electrodes. Due to the lower density of CNFs for solution B the electrolyte damages the metal underlayer in between the CNFs thus resulting in lower capacitance after 1000 cycles.

The above results indicate that the CNFs grown from all types of polymer-stabilized Pd nanocatalysts are vertically aligned similar to CNFs grown from a PVD deposited catalyst film. Moreover the density and weight of the CNFs grown from a PVP–Pd solution can be controlled by controlling the palladium contents in the PVP–Pd solution in the tested concentration range. Furthermore CNFs of different diameters can be grown from LauMA_*x*_-b-AEMA_*y*_/Pd solutions. Finally, the electrical characterization proves that electrical properties of all the CNFs are the same; however, the low capacitance in solution A is due to the lower density of CNFs in the footprint area.

The CNF growth performed at this temperature has created the opportunity to grow the CNFs directly on CMOS chips. The possibility of CNF growth at temperatures lower (350 °C) than CMOS temperature ensures their usage even if the CMOS temperature drops further.

## 4. Conclusions

Catalytic palladium NPs of various sizes were deposited on a silicon substrate via spin-coating using two types of highly stable and cost-effective polymer–Pd colloidal solutions. More precisely, PVP or methacrylate-based diblock copolymers of the type LauMA_*x*_-b-AEMA_*y*_ were employed as steric stabilizers for palladium NPs in organic solvents. Vertically aligned CNFs were synthesized on these palladium nanocatalysts using the direct plasma enhanced CVD technique at different temperatures ranging from 550 °C down to 350 °C, which can be considered as a CMOS-compatible temperature. The SEM characterization had shown that the CNFs grown at low temperature were vertically aligned similar to those generated at high temperature. The capacitive behavior and long cyclability had proven not only the suitability of the as-grown CNFs as electrodes for supercapacitors but also that the CNFs grown at low and high temperatures had similar electrical behavior. We have demonstrated a CMOS compatible process to build efficient heat sink, interconnects or supercapacitor electrodes directly on the chip based on vertically aligned CNFs as the active material.
